# Spasmodic dysphonia: the need for a combined neurological and phoniatric approach

**DOI:** 10.1007/s00702-024-02868-x

**Published:** 2024-12-24

**Authors:** Dirk Dressler, Bruno Kopp, Lizhen Pan, Andrew Blitzer, Fereshte Adib Saberi

**Affiliations:** 1 Movement Disorders Section, Department of Neurology, Hannover Medical School, Carl-Neuberg-Str. 1, 30625 Hannover, Germany; 2 Neurotoxin Research Center, Department of Neurology, Tongji University Medical School, Shanghai, China; 3 Department of Neurology, Hannover Medical School, Hannover, Germany; 4 Department of Otolaryngology-Head and Neck Surgery, Columbia University College of Physicians and Surgeons, New York, NY USA; 5 IAB - Interdisciplinary Working Group for Movement Disorders, Hamburg, Germany

**Keywords:** Spasmodic dysphonia, Dystonia, Abductor type, Adductor type, Mixed type, Essential tremor, Parkinsonian syndromes, Family history

## Abstract

Spasmodic dysphonia (SD) is now generally considered to be a task-specific focal dystonia. For the first time, we wanted to explore the relationship between SD and dystonia from a combined neurological and phoniatric perspective. For this, we studied 115 patients with non-psychogenic SD by a combined neurological and phoniatric evaluation. Onset of SD was 49.7 ± 19.0 (6–68) years. The female/male ratio was 2. 63% had additional dystonia manifestations (cervical dystonia 35%, arm dystonia 15%, blepharospasm 11%, oromandibular dystonia 11%, writer’s cramp 11%, pharyngeal dystonia 10%, generalised dystonia 4%, axial dystonia 2%, spasmodic dyspnoea 2% and segmental dystonia 1%). 71% occurred before, 25% after and 4% together with SD. 17% had a family history of dystonia and 6% a history of exposure to dopamine receptor blocking agents. 41% had mixed SD (SD-M), 31% abductor SD (SD-AB) and 28% adductor SD (SD-AD). SD-M was significantly correlated with additional dystonia manifestations and tremulous SD. No patient showed essential tremor or Parkinsonian syndromes. Two third of SD patients have additional dystonia manifestations and one fifth have a family history of dystonia, considerably more than previously described. In half of all patients, SD starts with non-SD dystonia. Our combined approach revealed a high prevalence of SD-M associated with frequent additional dystonia manifestations including dystonic tremor and a family history of dystonia. Patients presenting with SD should be evaluated for additional dystonia manifestations and dystonia patients should be evaluated for SD. Relevant coexistence of essential tremor and Parkinsonian syndromes cannot be confirmed.

## Introduction

Verbal communication can be disturbed by language disorders (aphasias) and by speech disorders (dysphonias). In dysphonias, the larynx and its neuronal control system is impaired. In spasmodic dysphonia (SD), the mechanics of the larynx are intact and dysarthria due to cerebellar incoordination, central and peripheral paresis is absent. The neuronal control of the larynx is impaired on a central level, probably involving the basal ganglia (Walter et al. 2014) making SD a form of dystonia, hence, its alternative name laryngeal dystonia. As SD only occurs, when the larynx muscles are activated for phonation, it is a task-specific dystonia. In summary, SD may be defined as involuntary muscle contractions of laryngeal muscles on phonation. With a prevalence of 24.7 per million population, it is the eighth most common form of dystonia (Dressler et al. 2022). The SD population has an age of 60.4 ± 17.5 years with a female preponderance of 60%, also typical for other idiopathic dystonias (Dressler et al. 2022). 85% of SD patients receive botulinum toxin therapy, 15% prefer other therapies or do not receive any treatment (Dressler et al. 2022).

Depending on the particular laryngeal muscle involvement pattern, three basic forms of SD can be distinguished. In the adductor form (SD-AD), the glottis is narrowed and the speech becomes strained, strangled and choppy with voice breaks and with words cut off or difficult to start. SD-AD is reported to be the most common form of SD. In the abductor form (SD-AB), the glottis is opened and the speech becomes weak, breathy and whispering. SD-AB is believed to be the second most common form of SD. In the mixed form (SD-M), elements of both, SD-AD and SD-AB, occur (Sulica 2004). Often tremor of the vocal cords is generated. As with other forms of dystonia, tricks, such as speaking with a high pitch, singing, laughing, crying or whispering may be used. Stress often aggravates the symptoms, whereas anxiolytics such as sedatives and alcohol reduces them (Sulica 2004).

When dystonia is classified as focal, it is frequently not entirely focal. For example, approximately 40% of patients with idiopathic cervical dystonia have additional dystonic manifestations in six different body parts producing blepharospasm, oromandibular dystonia, writer's cramp, arm dystonia, spasmodic dysphonia and axial dystonia (Dressler et al. 2022). The same might be true for SD.

For the first time, we wanted to study the full spectrum of SD’s presentation, including its clinical subforms, its additional dystonic manifestations, its patient demographics and its hereditary and aetiological aspects by applying a combined neurologic and phoniatric evaluation according to current movement disorder standards.

## Methods

### Design

This study is a combined prospective and retrospective study of a large cohort of patients with SD. Based on a formal collaboration, all patients were evaluated in the Movement Disorders Section and in the Department of Phoniatry of Hannover Medical School.

### Patients

Patient inclusion criteria consisted of (1) diagnosis of SD. (2) availability of complete data set of all pre-defined study parameters. Exclusion criteria included the diagnosis of psychogenic SD. Patients were included consecutively into this study until the pre-set number of 115 patients was reached.

### Study parameters

The study parameters are shown in Table 1. Patient demographics described the patient's sex, the patient's age at study inclusion and the patient's age at SD onset. First contact described, whether the patient was first seen in our institution by neurologists in the Movement Disorders Section or by phoniatricians in the Department of Phoniatry. Additional dystonia manifestations were identified in a specialised neurological examination. The patient’s family history of dystonia derived from personal examination of family members, diagnoses by other physicians and signs and symptoms provided by the patient. The level of diagnostic accuracy may, thus, best be described as 'probable'. Exposure to dopamine receptor blocking agents prior to SD onset was also documented.

**Table 1:** Study parameters with their dimensions

Parameter	Definition	Dimension
Patient age	Patient's age at time of inclusion in this study	Years
Patient age at onset	Patient's age at time of SD onset	Years
Patient sex	Patient's sex	Male/female
Observation period	Time between first contact with patient and study inclusion	Years
Initial contact	First contact with study centre	Neurologists/phoniatricians
SD-ADD	Spasmodic dysphonia, adductor type	Yes/no
SD-ABD	Spasmodic dysphonia, abductor type	Yes/no
SD-M	Spasmodic dysphonia, mixed type	Yes/no
ADM	Additional dystonia manifestations	Yes/no (description)
Age at ADM onset	Patient's age at ADM onset	Years
Family history of dystonia	Patient's family history of dystonia	Yes/no (description)
History of DRBA	Exposure to dopamine receptor blocking agents before SD onset	Yes/no (description)

### Statistics

All averages are given as means ± standard deviation. Statistical significance was set to *p* ≤ 0.05.

### Ethical approval

The study was performed under the regulations of the local ethical committee of Hannover Medical School.

## Results

Table 2 gives an overview about the main findings of our study. The differentiation between the different SD subtypes is shown in Table 3.

**Table 2:** Spasmodic dysphonia. Overview

Age at onset	49.9 ± 19.1 years (min. 6, max. 68)
Prevalence	24.7 per 1 million population
Female/male ratio	2.0
Family history of dystonia	17%
Family history of spasmodic dysphonia	3%
Additional dystonia manifestations	63% Cervical dystonia (35%) Arm dystonia (15%) Blepharospasms (11%) Oromandibular dystonia (11%) Others (28%)
Spasmodic dysphonia mixed type	41%
Spasmodic dysphonia abductor type	31%
Spasmodic dysphonia adductor type	28%

**Table 3:** Spasmodic dysphonia and its subforms

Parameter	SD-ALL	SD-AB	SD-AD	SD-M	Remarks
Frequency [%]	100	31	28	41	
Patient age at SD onset (mean ± sd) [y]	49.9 ± 19.1	50.9 ± 19.7	51.1 ± 14.7	47.8 ± 21.1	F (2,112) = 0.38, p = 0.68
Female/male ratio [n]	2.0	4.1	1.5	1.6	χ2 (2) = 4.43, p = 0.11
Additional dystonia manifestations [%]	63	58	57	95	χ2 (2) = 13.29, p = 0.001*
Family history of dystonia [%]	17	23	11	25	χ2 (2) = 2.03, p = 0.36
Tremolous SD [%]	14	6	0	30	χ2 (2) = 17.16, p < 0.001*
History of exposure to dopamine receptor blocking agents [%]	6	6	11	5	χ2 (2) = .88, p = 0.64

sd standard deviation, SD spasmodic dysphonia, SD-ALL patients with all forms of spasmodic dysphonia, SD-ABD patients with abductor form of spasmodic dysphonia, SD-ADD patients with adductor form of spasmodic dysphonia, SD-M patients with mixed form of spasmodic dysphonia

*Statistically significant

### All SD patients

Altogether, 115 patients with SD were included in this study. Their age was 69.3 ± 16.3 years and their SD manifestation age was 49.7 ± 19.0 years ranging from 6 to 68 years. 77 (67%) of them were female, 38 (33%) male, i.e., their female/male ratio was 2.0. The observation period, i.e., the period between the first contact with the patient in our institution and the time of the study, was 10.5 ± 4.9 years. 65 (56%) patients were initially seen in the Movement Disorders Section and 50 (44%) in the Department of Phoniatry.

42 (37%) patients had isolated SD. 73 patients (63%) had altogether 132 additional dystonia manifestations. Of those, 35% were cervical dystonia, 15% arm dystonia, 11% blepharospasm, 11% oromandibular dystonia, 11% writer's cramp, 10% pharyngeal dystonia, 4% generalised dystonia, 2% axial dystonia, 2% spasmodic dyspnoea and 1% segmental dystonia. Segmental dystonia was defined as dystonia occurring in two or more contiguous regions of the body. Pharyngeal dystonia was defined as involuntary activation of pharyngeal muscles, either occurring spontaneously or action-induced. Spasmodic dysphonea was defined as involuntary activation of pharyngeal, laryngeal and respiratory muscles when breathing is attempted (Zwirner et al. 1997). When there were additional dystonia manifestations, 53 (71%) occurred before SD, 18 (25%) after SD and 3 (4%) together with SD. Altogether, in 52 (45%) of all SD patients the initial symptomatology is non-SD dystonia.

20 patients (17%) had a family history of dystonia with 23 dystonia manifestations. Of those, 39% had cervical dystonia, 26% arm dystonia, 13% spasmodic dysphonia, 9% generalised dystonia, 4% blepharospasm, 4% oromandibular dystonia and 4% writer’s cramp. 7 patients (6%) had received dopamine receptor blocking agents before onset of SD, including classic and atypical neuroleptics, selective serotonin reuptake inhibitors and calcium channel blocking agents. 4 patients (3%) had a history of perinatal brain damage, 1 patient (1%) had cerebral leukaemia. No patient had signs of neurodegenerative disorders, such as Parkinsonian syndromes, or other movement disorders including essential tremor.

### SD-M

47 patients (41%) had SD-M. Their SD manifestation age was 47.8 ± 21.1 years. Their female/male ratio was 1.67. 95% had additional dystonia manifestations, 25% had a family history of dystonia, 30% had tremulous SD, 9% had perinatal brain damage and 2% had tardive aetiology.

### SD-AB

36 patients (31%) had SD-AB. Their SD manifestation age was 50.9 ± 19.7 years. Their female/male ratio was 4.1. 58% had additional dystonia manifestations, 23% had a family history of dystonia, 6% had tremulous SD and 6% had a tardive aetiology.

### SD-AD

32 patients (28%) had SD-AD. Their SD manifestation age was 51.1 ± 14.7 years. Their female/male ratio was 1.5. 57% had additional dystonia manifestations, 11% had a family history of dystonia and 11% had a tardive aetiology. None of them had tremulous SD.

## Discussion

### General

With a sample size of 115 SD patients, our study is one of the largest studies of its kind. It is, so far, the only study focusing on SD and its relationship with other neurological symptoms of dystonia.

### Patient demographics

**Fig. 1:**
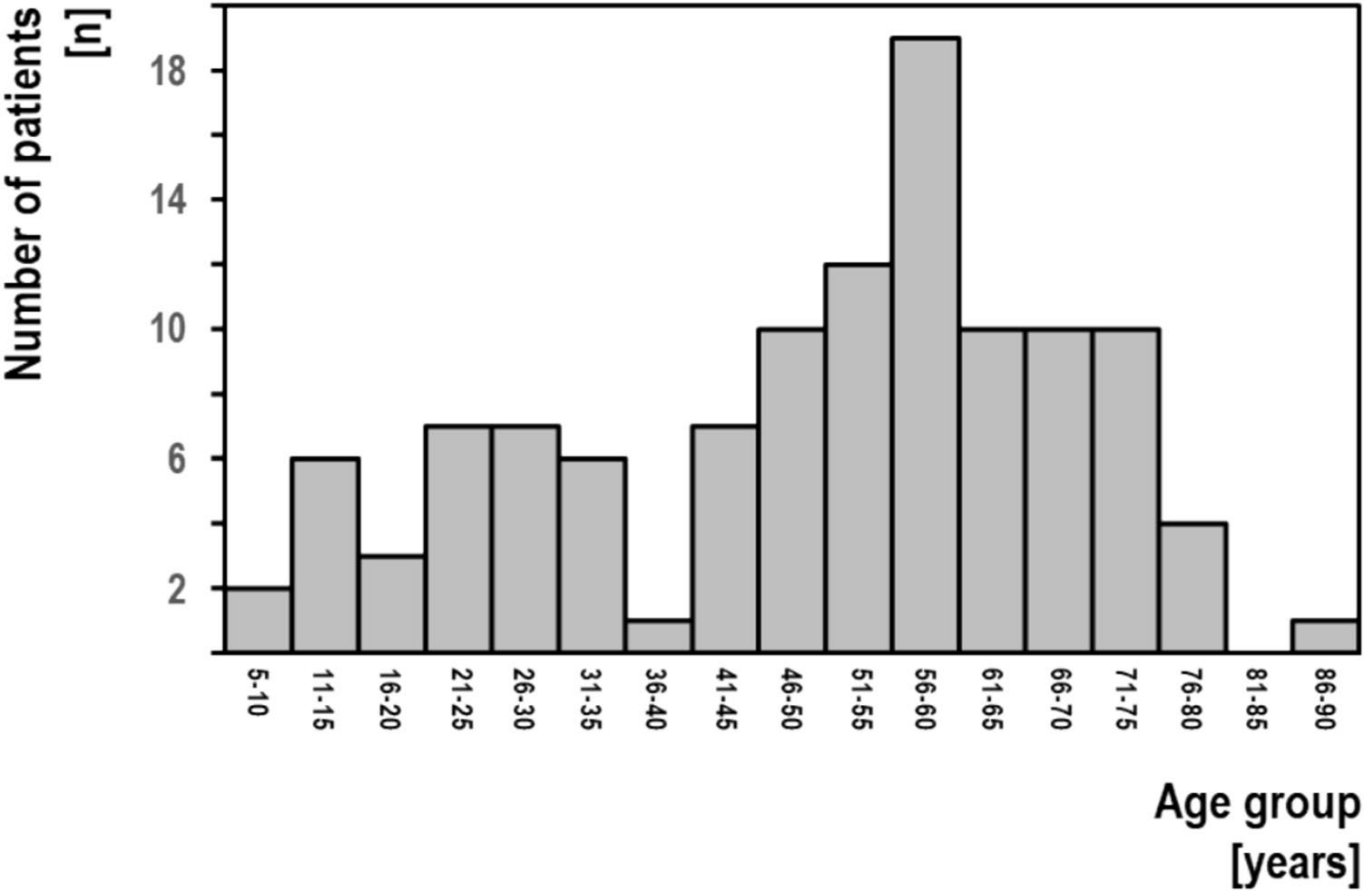
Onset of spasmodic dysphonia

Our study shows a patient age at SD onset of 49.9 ± 19.1 years with a range of 6–68 years. This is similar to previously reported data ranging from 44 to 50 years (Tanner et al. 2011; Aronson et al. 1968; Schweinfurth et al. 2002; Brodnitz 1976). Obviously, the patient age at SD onset is different from the patient age at study inclusion and both data shouldn’t be confused. In our group, the patient age at study inclusion was 69.3 ± 16.3 years. As shown in Fig. 1, there are a number of patients with early onset. Some of these patients presented with perinatal brain damage, others might represent cases with strong genetic aetiology known to manifest early.

In our study, SD prevalence was reported as 24.7 per million population, making it the eighth most common form of dystonia with 3% of all dystonia manifestations. Claims of SD being the third most common form of focal dystonia are over-estimates and obviously blurred by methodological flaws (Castelon-Konkiewitz et al. 2002).

Our study also shows a strong female predominance of 2.0. Previously reported female/male ratios range from 1 to 7 (Nutt et al. 1988; Schweinfurth et al. 2002, Blitzer et al. 2018, Adler et al. 1997). Our figure is in line with a similar female predominance recently reported in other idiopathic focal dystonias (except for musician’s dystonia) (Dressler et al. 2022).

A family history of dystonia was identified in 17% of our SD patients including SD and a large variety of other focal, segmental or generalised dystonia manifestations. For SD, 3% of of our SD patients had a family history. Previous data on the family history of dystonia are sparse, suggesting a frequency of 12% for all forms of dystonia (Blitzer et al. 2017), a 'very low' frequency for SD (Ludlow 2010) or a non-existent one for SD (Schweinfurth et al. 2002). Reasons for these discrepancies, especially for non-SD dystonias, are likely to be patient evaluations performed by experts lacking up-to date expertise in movement disorders, especially dystonia.

### Clinical symptomatology

Descriptions of the relative frequency of SD subforms in the literature are surprisingly vague. Unanimously, SD-AD is believed to be by far the most common form of SD with a frequency of around 80% to 90% (Ludlow 2010, Edgar et al. 2001; Hyodo et al. 2021, Blitzer et al. 2017), whereas SD-AB is thought to be rare (Ludlow 2010) with frequencies of around 15% (Edgar et al. 2001, Blitzer et al. 2017). Figures for SD-M are not readily available. Our study revealed contrasting relative frequencies of SD-AB of 31%, of SD-AD of 28% and of SD-M of 41%. Reasons for this discrepancy are not immediately clear. First of all, our combined neurological and phoniatric approach retrieved an entirely different patient population with much higher frequencies of additional dystonia manifestations and family histories of dystonia than previously reported. Mainly, however, this observation seems to reflect previous terminological confusion, especially with respect to essential tremor, which is a separate clinical entity characterised by isolated tremor not associated with any dystonic elements, occurring on action, but not being task-specific and predominantly affecting the upper extremities. If voice tremor is misdiagnosed as essential tremor, as it was in the past, these patients were excluded from the spectrum of SD. In our study population, 30% of all SD-M patients had dystonic voice tremor, whilst essential voice tremor could not be detected. 6% of our SD-AB patients presented with voice tremor. Whether dystonic voice tremor without any tonic spasmodic dysphonation may exist, is unclear. Considering our combined observations, we suggest that SD is classified according to its tonic dystonia elements as SD-AD, SD-AB and SD-M. Dystonic tremor may be superimposed. Pure dystonic tremor of the larynx may exist.

Whereas in our study 63% of all SD patients had additional dystonia manifestations, this frequency was previously reported with around 16% (Blitzer et al. 1998). Around 30% of SD patients are reported to have additional vocal tremor (Ludlow 2010, Tanner et al. 2011; White et al. 2011; Schweinfurth et al. 2002). This tremor was believed to represent the co-existence of SD and essential tremor. In our study, the frequency of tremulous SD was 14%, similar to previously reported figures. However, it was not caused by essential tremor. Instead, it reflected dystonic tremor, as all patients with tremulous SD had dystonia manifestations elsewhere in the body and all patients with tremulous SD had tonic dystonic laryngeal muscle involvement and some had gestes antagonistes or other sensory tricks.

Confusion about the nature of tremor syndromes is common and reflects classificatory deficits from times, before the dystonia concept was developed and the role of dystonic tremor became apparent. Although we are not challenging the existence of ET affecting laryngeal muscles, we believe that this is rare. The same might be true for Parkinsonian tremor. For SD patients initially diagnosed in our department, there could be a possible bias favoring the prevalence of dystonia, as our main expertise is perceived by referral partners to be dystonia and botulinum toxin therapy. However, half of the SD in our study were initially seen in the Department of Phoniatry, where this bias did not exist.

## Conclusions

In our study, for the first time, an entire cohort of SD patients was evaluated by an interdisciplinary collaboration of neurologists and phoniatricians. This collaboration revealed a clinical symptomatology considerably different than previously reported. There was a substantially higher frequency of SD-M, of additional dystonia manifestations and of family history of dystonia. This confirms the view that SD is part of the dystonic syndrome. It also suggests to evaluate SD in an interdisciplinary setting with a collaboration of neurologists and phoniatricians. Phoniatricians should involve neurologists to better understand the entire dystonic involvement of their SD patients and neurologists should involve phoniatricians to evaluate involvement of the larynx and the pharynx in their dystonia patients. Identification of additional dystonic manifestations may be helpful to confirm the diagnosis dystonic SD, when the diagnosis of a laryngeal dysfunction is unclear. Involvement of additional medical specialties may be helpful to improve treatment results.

### Outlook

Future research should provide epidemiological data on the frequency symptomatic SD such as Parkinsonian syndromes and of essential tremor. Technical devices and standardised evaluation procedures and improved diagnostic criteria would be helpful to better differentiate SD subforms, which should—in return- improve the outcome of botulinum toxin therapy.

## Data Availability

Raw data are available upon reasonable request.
